# Determinants of overall knowledge of and attitudes towards HIV/AIDS transmission among ever-married women in Pakistan: evidence from the Demographic and Health Survey 2012–13

**DOI:** 10.1186/s12889-019-7124-3

**Published:** 2019-06-21

**Authors:** Sarosh Iqbal, Sidra Maqsood, Asma Zafar, Rubeena Zakar, Muhammad Zakria Zakar, Florian Fischer

**Affiliations:** 1 Institute of Social and Cultural Studies, University of the Punjab, Lahore, Pakistan; 2 School of Public Health, Bielefeld University, Bielefeld, Germany

**Keywords:** HIV/AIDS, Comprehensive knowledge, Positive attitudes, Pakistan, PDHS, 2012–13

## Abstract

**Background:**

HIV/AIDS has emerged as a serious public health issue across the globe, and particularly in developing countries. Comprehensive knowledge and positive attitudes are cornerstones for the prevention, control and treatment of HIV/AIDS. However, there are various misconceptions associated with HIV/AIDS transmission, which lead to negative attitudes towards people living with AIDS. The present study aims to explore the effects of these determinants, related to socio-demographic characteristics and autonomy, on women’s overall knowledge and attitudes regarding HIV/AIDS in Pakistan.

**Methods:**

Secondary data analysis was carried out using the national representative dataset of the 2012–13 Pakistan Demographic and Health Survey. A series of questions related to HIV/AIDS was asked of 13,558 ever-married women aged 15–49 years to assess respondents’ knowledge regarding modes of HIV/AIDS transmission and preventative measures, as well as their attitudes towards people living with HIV/AIDS. Descriptive and bivariate statistics were used to identify associations with socio-demographic and autonomy-related variables. Furthermore, bivariate and multivariate logistic regression analyses were performed to assess the association between multiple factors and overall HIV/AIDS knowledge as well as attitudes towards people living with AIDS.

**Results:**

The results show that only 42% of Pakistani women have heard about HIV/AIDS. Amongst these women, the majority (68%) have good overall knowledge of HIV/AIDS and more than 55% have positive attitudes towards people living with AIDS. Furthermore, women residing in urban areas, having at least secondary-level education, with high autonomy, belonging to the richest wealth quintile and having exposure to mass media had high overall knowledge and positive attitudes towards people living with AIDS.

**Conclusion:**

The findings of this research support the relevance of women’s autonomy, education and exposure to mass media, particularly in rural areas of Pakistan, to address the lack of knowledge and eliminate various myths and stigmatisation of people living with HIV/AIDS. Furthermore, it reveals a need to increase focused and targeted interventions to enhance women’s knowledge and positive attitudes towards people living with HIV/AIDS. In this regard, the media can play a proactive role to gauge wider audience in creating awareness and eradicating the myths and misconceptions regarding HIV/AIDS.

**Electronic supplementary material:**

The online version of this article (10.1186/s12889-019-7124-3) contains supplementary material, which is available to authorized users.

## Background

Human Immunodeficiency Virus (HIV) causing Acquired Immunodeficiency Syndrome (AIDS) has emerged as a serious public health issue across the globe, and particularly in developing countries. The latest UNAIDS global statistics reported that 36.9 million people are HIV positive, including 35.1 million adults (15+ years) and 1.8 million children (< 15 years) [1, 2]. Amongst these, 1.8 million people are newly infected [1]. Overall, the number of HIV-positive adult women (15+ years) is higher, i.e. 17.8 million, constituting 48.5% of the total HIV-infected population [1]. Since the start of the epidemic, the number of deaths from AIDS-related illness has exceeded 35 million, while approximately 21 million people had received antiretroviral therapy by 2017 [1, 2].

Asia and the Pacific region are home to 5.1 million HIV-infected people, with an estimated prevalence of 0.2% [3], including 1.82 million adult women and 1.65 million adult men (15+ years) [4]. Recent data from UNAIDS and the National AIDS Control Programme highlighted that there are 1.3 million HIV-infected people in Pakistan [5, 6], but only 22,333 HIV-positive are registered and currently only 12,046 are receiving antiretroviral therapy [6]. A series of HIV surveillance results indicate that the epidemic is already established, particularly in risk groups [7], and this requires immediate attention.

In the context of the traditional Muslim society of Pakistan, prevention and response to the growing epidemic of HIV/AIDS is quite challenging. Due to the stigmatisation [8] and risky behaviours associated with transmission of HIV/AIDS [9], there are various misconceptions and myths [10, 11] attributed to conventional cultural beliefs and practices. Stigmatisation related to HIV/AIDS highlights not only deficiencies in general knowledge but also negative and unacceptable attitudes towards people living with HIV/AIDS (PLWHAs) [9]. Such discriminatory behaviours inhibit people from accessing the available HIV/AIDS prevention and treatment options [12], due to increased fear of being stigmatised. The literature revealed that inaccurate knowledge about the transmission of HIV/AIDS contributes to people’s stigmatising statements and negative attitudes [13], thus limiting the social support for PLWHAs [14].

Previous research suggests that women’s knowledge about HIV/AIDS is lower than men’s [15–17]. Therefore, women’s risk of contracting HIV/AIDS becomes heightened, coupled with various contributing factors such as low literacy, limited access to preventive health services, low autonomy, sexual and emotional violence and legal disenfranchisement [18].

Given the context above, there is a need for renewed attention and additional research to understand the effects of various determinants related to women’s socio-demographics and autonomy in shaping their overall knowledge and attitudes towards PLWHAs in Pakistan. Various studies have been conducted so far in local settings, focusing particularly on knowledge, attitudes and practices around HIV/AIDS amongst high-risk groups [19–22]. Moreover, what little evidence is available in Pakistan stems from studies with a small sample size about the general population’s knowledge and attitude on HIV/AIDS [23–25], along with a few studies comparing women’s and men’s knowledge and attitudes around HIV/AIDS, focusing on socio-demographic factors only [16, 26]. Hence, this research will be a value-addition, aiming to explore the effects of various determinants related to socio-demographic factors and autonomy on women’s overall knowledge about HIV/AIDS and attitudes towards PLWHAs in Pakistan. This research is highly pertinent considering the significance of the Global AIDS Monitoring indicators (2018), which emphasise regular reporting against the “2020 fast-track commitments and expanded targets to end AIDS” [27]. Within these global indicators, commitments 4 and 5 specify eliminating the discrimination against women, girls and PLWHAs and ensuring that 90% of young people have the knowledge they need to protect themselves from HIV by 2020 [27].

### Theoretical framework

**Fig. 1:**
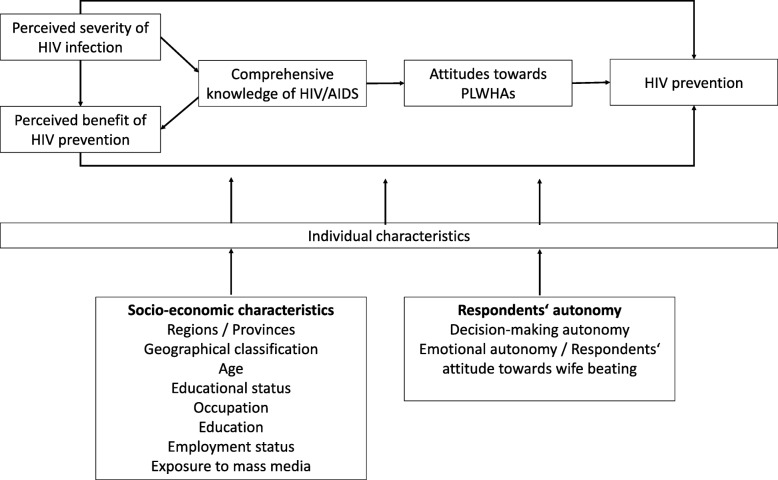
Theoretical framework

Considering the research objectives for exploring the factors related to HIV/AIDS prevention knowledge and attitude, the Social Cognitive Theory and Information Motivation Behavioural Skills (IMB) theory were adapted. Social Cognitive Theory, an advanced form of Bandura’s social learning theory, presented the most comprehensive principal to understand the behavioral change [28, 29]. This theory emphasised that acquiring the disease’ knowledge, i.e. what it is, how it is transmitted and how it can be prevented, leads to behavioral change, coupled with intentions to perform preventive measures and attain desired outcomes (protection from the disease) [28, 30]. It indicated towards individuals’ perceived severity of risks and capability to perform certain behaviour to avoid negative attitudes, resulting from fear of being stigmatised [28, 30, 31]. Similarly, IMB theory, which was originally developed to explore the determinants of HIV risk and preventive behaviour, endorsed that health related information, motivation and behavioural skills are essential to perform health behaviours [32–34]. The IMB theory also suggested that perception of HIV risks not only inclined the individual to acquire accurate HIV knowledge, but also motivate to equip with necessary skills for HIV preventive behaviours [29, 35]. Altogether, the comprehensiveness of these theories made them most significant behavioural change facilitators, thus was employed in multiple studies and found moderately effective in HIV prevention [28–31, 36–38]. Hence, recognising significance of above theories, this research demonstrates the importance of relevant HIV prevention knowledge and attitudes, in the presence of perceived severity of risks/infection and various moderating factors to achieve desired benefits of HIV prevention. These moderating factors include women’s socio-economic and autonomy related characteristics, affecting HIV prevention and high risk behaviors to fight against HIV/AIDS. An illustration of the theoretical framework is given in Fig. 1.

## Methods

### Data source

The study is based on a secondary analysis using nationally representative data from the Pakistan Demographic and Health Survey (PDHS) 2012–13 [39]. The PDHS 2012–13 is the third survey conducted as part of the MEASURE DHS (Demographic and Health Survey) international series with the financial support of USAID. The National Institutes of Population Studies (NIPS) completed the PDHS with technical support from ICF International and the Pakistan Bureau of Statistics. The PDHS 2012–13 is the largest publicly available household dataset in Pakistan to have collected information on variables related to HIV/AIDS awareness and attitudes among the general population. The cross-sectional study used a two-stage cluster sampling technique for data collection. During the first stage, sampling areas of 248 urban and 252 rural units were selected; in the second stage, 14,000 households (6944 from urban areas and 7056 from rural areas) were selected through systematic random sampling. The fieldwork was completed between October 2012 and March 2013, during which time a total of 20 field teams, each comprising a supervisor, a field editor, one male and three female interviewers, collected data. These teams were supervised by quality-control interviewers, field coordinators and senior NIPS team members. Along with the fieldwork, the data processing, including editing and entry of completed questionnaires, was initiated simultaneously. Moreover, all data was entered twice using the CSPro computer package within the NIPS office for 100% verification [39].

The PDHS 2012–13 used four types of questionnaires for data collection, consisting of: a household questionnaire, a women’s questionnaire, a men’s questionnaire, and a community questionnaire. The standard women’s questionnaire, used in this analysis, was administered to 13,558 ever-married women of reproductive age (15–49 years) through face-to-face interviews, with a response rate of 93% [28]. A series of questions related to overall knowledge and attitudes around HIV/AIDS were also part of the standard women’s questionnaire to assess respondents’ knowledge regarding modes of HIV/AIDS transmission and the ways in which HIV/AIDS can be prevented, as well as their attitudes towards PLWHAs [39].

### Variables

#### Outcome variables

Women’s overall knowledge about HIV/AIDS and their attitudes towards PLWHAs are the outcome variables for this research. Both variables were inferred from a series of questions used in the PDHS 2012–13 questionnaire [39], consistent with previous similar studies [16, 40] and also aligned with the global AIDS monitoring indicators of 2018 [27]. All the women who had ever heard of HIV/AIDS further responded to questions on their knowledge and attitudes towards PLWHAs. The construction of both variables is published elsewhere [40].

Women’s overall knowledge on HIV/AIDS was inferred from five questions, including knowing about the two most common methods to prevent HIV/AIDS infection: 1) consistent condom use and 2) limiting the number of sexual partners to one uninfected partner who is faithful. Additionally, it was assessed whether the respondents were able to reject three common misconceptions about HIV/AIDS: 3) a person can get HIV from a mosquito bite, 4) a person can get HIV by sharing a meal with an infected individual, and 5) a healthy-looking person can have HIV/AIDS. The answer categories for the above questions were “yes”, “no”, and “don’t know”. Hence, for this research, the incorrect and “don’t know” responses were re-coded as 0, while correct responses were re-coded as 1. Thus, the score for women’ overall knowledge on HIV/AIDS ranged from 0 to 5; where a woman who answered all five questions incorrectly had a score of 0 and a woman who answered any of the five questions correctly got a score between 1 and 5. The mean value was taken as a cut-off value for dichotomisation of women’ overall knowledge on HIV/AIDS scores into high vs. low knowledge. According to this, women who had a score of 3–5 were coded as having high HIV/AIDS knowledge, while the women who had a score of 0–2 were coded as having low knowledge.

Similarly, women’s attitudes towards people living with HIV/AIDS was measured through the following four questions: 1) “Would you want to keep a family member’s HIV infection a secret?”, 2) “Would you care for a relative who is infected with HIV?”, 3) “Would you buy vegetables from a vendor who has AIDS?”, and 4) “Should a female teacher infected with HIV be allowed to continue teaching in school?” The answer categories for these questions were “yes”, “no” and “don’t know”. Here, the “no” and “don’t know” responses were also re-coded as 0, having a negative attitude, and “yes” responses were re-coded as 1, having a positive attitude towards PLWHAs. Thus, the score for each woman’s attitude towards PLWHAs was computed for this analysis, ranging from 0 to 4, where a total score of 0 represented a negative attitude and a score of 1–4 indicated a positive attitude in any of the four scenarios. Again, the mean value was taken as a cut-off value for dichotomisation of scores into positive vs. negative attitudes. Thus, the women, who acquired 3–4 score, were coded as having a positive attitude, while the women, who had a score of 0–2 were considered as having a negative attitude towards PLWHAs.

#### Autonomy variables

Women’s autonomy is measured through two variables: a) their overall participation in multiple household decisions, and b) emotional autonomy, referring to their attitudes towards domestic violence, highlighting their opinions (agreement or disagreement) on wife beating. The construction of both these variables is published elsewhere [40, 41].

Women’s autonomy in terms of participation was inferred from the five questions: 1) “Who (in your family) usually decides how to spend your earnings?”, 2) “Who usually decides on making large household purchases?”, 3) “Who usually decides on your visits to family or relatives?”, 4) “Who usually decides on your healthcare?”, and 5) “Who usually decides what to do with your husband/partner’s earnings?” Possible responses to the first three autonomy questions were: “respondent alone”, “husband/partner alone”, “respondent and husband/partner jointly”, “respondent and other person”, “someone else”, “family elders” or “others”. Possible responses to who usually decides what to do with the husband/partner’s earnings were “respondent alone”, “husband/partner alone”, “respondent and husband/partner jointly”, “respondent and other person”, “someone else”, “family elders”, “the husband/partner does not bring in any money” or “other”. For this study, responses to the above autonomy questions were dichotomised into two categories: whether the woman has “a say at all” (either alone or jointly with her husband/partner or jointly with another person) coded as 1, or whether she has “no say at all” coded as 0 (in cases where the husband/partner, family elders or someone else makes the decision). This dichotomisation of autonomy/decision-making is consistent with previous research work done using the DHS datasets [40, 41]. Based upon these five binary household decision-making questions, the score for women’ autonomy was computed for each woman ranging from 0 to 5, where if she had no say in any of the five decisions, her total score was 0 and if she had a say in any of the five household decisions her total score ranged between 1 and 5. Further, the mean value was taken as a cut-off value for the dichotomisation of autonomy scores into high vs. low autonomy. Women who had scores of 2–5 were coded as having high autonomy, while women with scores of 0 or 1 were considered to have low autonomy.

Women’s emotional autonomy was assessed through their attitudes towards domestic violence (wife beating). PDHS asked the women about situations when sometimes a husband is annoyed or angered by things that his wife does. In the respondent’s opinion, is a husband justified in hitting or beating his wife in the following situations: 1) “If she goes out without telling her husband?”, 2) “If she neglects the children?”, 3) “If she argues with her husband?”, 4) “If she refuses to have sex with her husband?”, and 5) “If she burns the food?”. Response categories for the wife-beating questions were “yes”, “no”, or “don’t know”. For the purposes of this study, the “yes” and “don’t know” response categories were re-coded as 0, while “no” responses were re-coded as 1. Based upon the five questions above, the scores for women’s emotional autonomy (attitude towards wife beating) was computed as ranging from 0 to 5, where a woman with a score of 0 agreed with all five circumstances of wife beating, whereas a woman having score of 1–5 disagreed with wife beating under one or more of the five circumstances. Furthermore, the mean value (i.e. 3) was taken as a cut-off value for dichotomisation of emotional autonomy or attitudes towards wife beating into disagreement vs. agreement. Women who had a score of 3–5 were coded as having high emotional autonomy, disagreeing about wife beating situations, while women with a score of 0–2 were coded as having low emotional autonomy, agreeing with the wife beating circumstances. For the sake of this analysis, women’s agreement or disagreement on wife beating will likewise represent emotional autonomy.

#### Socio-demographic variables

Based on the existing literature and available data within the PDHS 2012–13, a number of socio-demographic variables were included in the analysis [39, 40]. These were: respondents’ region/province (Punjab, Sindh, Baluchistan, Khyber Pakhtunkhwa, Gilgit Baltistan, Islamabad), the geographical classification of their residence (urban/rural), respondents’ age (15–24 years, 25–34 years, 35 years and above), educational level of the women and their husbands (each was grouped into four categories: uneducated/no formal schooling, primary, secondary, higher education), occupation of respondents and their husbands (each was grouped into four categories: unemployed; working in professional/managerial positions, including sales & services; agriculture; and unskilled or manual/household workers) and respondents’ exposure to mass media, including newspapers, TV and radio to access information (yes/no). Moreover, a composite index of household wealth was grouped into five quintiles (richest, richer, middle, poorer, poorest), measured on the basis of household assets and ownership of a number of consumer items [42].

### Statistical analysis

IBM SPSS® version 21 was used for data analysis. Sampling weights were used. Descriptive statistics for the variables of socio-demographics, autonomy and comprehensive knowledge and attitudes towards PLWHAs were compiled, and frequency distributions and percentages were presented. Cross-tabulations and chi-square tests were performed to assess the significance. A significance level of *p* < 0.05 was chosen. Simple binary logistic regression was used to determine the association of the predictors with women’s overall knowledge of HIV/AIDS and their attitudes towards PLWHAs. Afterwards, a multiple logistics regression was conducted using only those variables that were found to be significantly associated with both outcome variables. Further regression models are presented in the Additional file 1.

## Results

### Socio-demographic characteristics

Table 1 indicates the socio-demographic characteristics of the 13,558 respondents (women of reproductive age, 15–49 years). The results show that the majority of women (57.5%) were from Punjab province and the fewest from Islamabad (0.5%). Most of the women were from rural areas (66.5%) and in the age group of 35 years and above (41.3%). More than 57% of respondents were found to be without any formal schooling and 71% were unemployed. However, of their husbands, more than 33% had attained secondary-level education and 47.4% were employed as unskilled workers. Around 72% of respondents had access to mass media and 20.7% belonged to the richest wealth quintile.

**Table 1:** Socio-demographics characteristics of women from PDHS 2012–2013 (*n* = 13,558)

Characteristics	n = 13,558	
	f	%
Regions/Provinces		
Punjab	7790	57.5
Sindh	3133	23.1
Baluchistan	568	4.2
Khyber Pakhtunkhwa	1908	14.1
Gilgit Baltistan	94	0.7
Islamabad	64	0.5
Geographical classification		
Urban	4536	33.5
Rural	9022	66.5
Respondents age		
15–24 years	2711	20.0
25–34 years	5253	38.7
35 years and above	5594	41.3
Respondents educational status		
No formal schooling	7736	57.1
Primary	2156	15.9
Secondary	2406	17.7
Higher	1260	9.3
Respondents occupation		
Unemployed	9623	71.0
Professional/Managerial	1332	9.8
Agriculture	1445	10.7
Unskilled workers	1157	8.5
Husbands educational status		
No formal schooling	4452	33.0
Primary	2199	16.3
Secondary	4572	33.8
Higher	2288	16.9
Husbands employment status		
Unemployed	347	2.6
Professional/Managerial	4569	33.7
Agriculture	2215	16.3
Unskilled workers	6426	47.4
Exposure to mass media		
Yes	9852	72.7
No	3705	27.3
Wealth index		
Richest	2804	20.7
Richer	2789	20.6
Middle	2700	19.9
Poorer	2676	19.7
Poorest	2589	19.1
Measures for women’s autonomy and justification towards wife beating, comprehensive knowledge and attitude on HIV/AIDS		
Respondents’ autonomy		
High autonomy	7141	55.3
Low autonomy	5783	44.7
Respondents’ attitude towards wife beating		
Disagree on wife beating	5003	36.9
Agree on wife beating	8537	63.1
Respondents’ ever heard of HIV/AIDS		
Yes	5675	41.9
No	7870	58.1
Respondents’ comprehensive HIV/AIDS knowledge		
High knowledge	3859	68.0
Low knowledge	1814	32.0
Respondents’ attitude towards PLWHAs		
Positive attitude	3128	55.2
Negative attitude	2538	44.8

The findings also highlighted that more than 55% of respondents have high autonomy, while 63% of women agreed with wife beating. Around 42% of the women had heard about HIV/AIDS and amongst these, 68% had high overall knowledge of HIV/AIDS and more than 55% had a positive attitude towards PLWHAs.

### Bivariate analyses

Table 2 shows the cross-tabulation of dependent variables, including women’s overall knowledge of HIV/AIDS and their attitudes towards PLWHAs with various socio-demographics and autonomy-related determinants.

**Table 2:** Relationship of women’s comprehensive HIV/AIDS knowledge and attitude towards PLWHAs with their SES and autonomy from PDHS 2012–2013 (*n* = 13,558)

Characteristics	Women’s comprehensive HIV/AIDS knowledge			Women’s attitude towards PLWHA		
	High knowledge %	Low knowledge%	*p*-value*	Positive attitude %	Negative attitude %	*p*-value*
Regions/Provinces						
Punjab	59.5	69.8	< 0.01	62.6	62.9	0.43
Sindh	27.3	17.2		24.3	23.9	
Baluchistan	9.3	11.1		9.9	9.8	
Khyber Pakhtunkhwa	2.6	1.2		1.9	2.4	
Gilgit Baltistan	0.2	0.2		0.2	0.3	
Islamabad	1.1	0.5		1.1	0.7	
Geographical classification						
Urban	60.2	44.8	< 0.01	56.2	54.1	0.11
Rural	39.8	55.2		43.8	45.9	
Respondents age						
15–24 years	14.6	19.0	< 0.01	16.9	15.1	< 0.01
25–34 years	44.8	41.6		45.8	41.2	
35 years and above	40.6	39.4		37.3	43.7	
Respondents educational status						
No formal schooling	20.6	34.5	< 0.01	21.4	29.6	< 0.01
Primary	16.9	23.8		17.2	21.4	
Secondary	36.2	31.0		35	34.0	
Higher	26.3	10.7		26.4	15.1	
Respondents occupation						
Unemployed	79.7	77.4	< 0.01	78.1	80.1	< 0.01
Professional/Managerial	14.3	11.6		15	11.5	
Agriculture	1.7	4.1		2.1	2.9	
Unskilled workers	4.3	6.8		4.8	5.5	
Husbands educational status						
No formal schooling	13.9	20.5	< 0.01	13.7	18.8	< 0.01
Primary	11.1	15.6		11.8	13.4	
Secondary	40.6	43.7		42.3	40.7	
Higher	34.4	20.2		32.3	27.1	
Husbands employment status						
Unemployed	2.1	2.4	< 0.01	2.3	2.0	< 0.01
Professional/Managerial	50.1	40.9		50.5	43.1	
Agriculture	8.5	10.8		8.2	10.3	
Unskilled workers	39.4	46.0		38.9	44.6	
Exposure to mass media						
Yes	94.6	89.7	< 0.01	94.5	91.3	< 0.01
No	5.4	10.3		5.5	8.7	
Wealth index						
Richest	48.7	26.6	< 0.01	45.1	37.5	< 0.01
Richer	27	33.5		27.2	31.1	
Middle	14.9	24.3		17.6	18.3	
Poorer	7.0	11.6		7.7	9.4	
Poorest	2.4	4.1		2.4	3.7	
Respondents’ autonomy						
High autonomy	64	58.9	< 0.01	62.8	62	0.53
Low autonomy	36	41.1		37.2	38	
Respondents’ attitudes towards wife beating						
Disagree on wife beating	80.2	69.9	< 0.01	80.1	72.9	< 0.01
Agree on wife beating	19.8	30.1		19.9	27.1	

* Chi-square analysis was applied

Women’s overall knowledge of HIV/AIDS was found to be high among women from Punjab and Sindh provinces, residing in urban areas, in the age group 25–34 years, having secondary education, exposure to mass media and belonging to the richest wealth quintile. Women with high autonomy and disagreement with wife beating also reported having high overall knowledge about HIV/AIDS.

Regarding women’s attitudes towards PLWHAs, the results revealed positive attitudes among women from Punjab and Sindh provinces, living in urban areas, 25–34 years of age, having secondary education, with exposure to mass media and belonging to the richest quintile. Moreover, positive attitudes were seen in the respondents having high autonomy and who disagreed with wife beating.

A statistically significant association (*p* < 0.05) of both outcome variables was seen with respondents’ age, educational status and the occupation of respondents and their husbands, exposure to mass media, household wealth quintile and respondents’ attitudes towards wife beating.

### Bivariate and multivariate logistic regression

Tables 3 and 4 show the bivariate and multivariate logistic regression analysis of women’s overall HIV/AIDS knowledge and attitudes towards PLWHAs related to various socio-demographic and autonomy-related factors.

**Table 3:** Bivariate and multivariate logistics regression of women’s comprehensive HIV/AIDS knowledge with their SES and autonomy from PDHS 2012–2013

Characteristics	Women’s comprehensive HIV/AIDS knowledge					
	Bivariate			Multivariate		
	OR	CI (95%)	*p*-value	AOR	CI (95%)	*p*-value
Regions/Provinces						
Punjab	1			1		
Sindh	1.86	1.61–2.15	< 0.01	1.53	1.30–1.81	< 0.01
Baluchistan	0.98	0.82–1.18	0.87	1.19	0.97–1.47	0.08
Khyber Pakhtunkhwa	2.46	1.55–3.91	< 0.01	3.59	2.20–5.85	< 0.01
Gilgit Baltistan	1.67	0.44–6.43	0.45	1.37	0.34–5.45	0.65
Islamabad	2.69	1.31–5.51	< 0.01	1.61	0.75–3.44	0.22
Geographical classification						
Rural	1			1		
Urban	1.86	1.66–2.08	< 0.01	1.04	0.89–1.21	0.61
Respondents age						
15–24 years	1			1		
25–34 years	1.39	1.19–1.64	< 0.01	1.32	1.11–1.56	< 0.01
35 years and above	1.34	1.14–1.57	< 0.01	1.26	1.05–1.52	0.01
Respondents educational status						
No formal schooling	1			1		
Primary	1.19	1.01–1.40	0.03	1.14	0.95–1.36	0.16
Secondary	1.95	1.69–2.25	< 0.01	1.56	1.31–1.86	< 0.01
Higher	4.08	3.39–4.92	< 0.01	2.59	2.05–3.27	< 0.01
Husbands educational status						
No formal schooling	1			1		
Primary	1.06	0.85–1.29	0.59	0.99	0.80–1.24	0.98
Secondary	1.37	1.17–1.61	< 0.01	1.05	0.88–1.26	0.56
Higher	2.52	2.11–3.01	< 0.01	1.16	0.94–1.44	0.17
Respondents occupation						
Unemployed	1			1		
Professional/Managerial	1.19	1.01–1.42	0.04	1.09	0.91–1.32	0.35
Agriculture	0.41	0.29–0.57	< 0.01	0.64	0.44–0.94	0.02
Unskilled workers	0.61	0.47–0.77	< 0.01	0.91	0.69–1.19	0.47
Husbands occupation						
Unemployed	1	31				
Professional/Managerial	1.39	0.95–2.03	0.09	–	–	–
Agriculture	0.89	0.59–1.34	0.58	–	–	–
Unskilled workers	0.97	0.66–1.42	0.87	–	–	–
Exposure to mass media						
No	1			1		
Yes	2.01	1.64–2.47	< 0.01	1.25	0.99–1.58	0.06
Wealth index						
Poorest	1			1		
Poorer	1.02	0.72–1.45	0.90	0.96	0.66–1.41	0.85
Middle	1.04	0.75–1.44	0.81	0.96	0.67–1.39	0.84
Richer	1.36	0.98–1.87	0.06	0.98	0.68–1.43	0.95
Richest	3.09	2.24–4.26	< 0.01	1.62	1.09–2.41	0.01
Respondents autonomy						
Low autonomy	1			1		
High autonomy	1.24	1.10–1.39	< 0.01	1.15	1.01–1.32	0.03
Respondents attitudes towards wife beating						
Agree on wife beating	1			1		
Disagree on wife beating	1.75	1.54–1.98	< 0.01	1.18	1.02–1.37	0.02

**Table 4:** Bivariate and multivariate logistics regression of women’s attitude towards PLWHAs with their SES and autonomy from PDHS 2012–2013

Characteristics	Women’s attitude towards PLWHAs					
	Bivariate			Multivariate		
	OR	CI (95%)	*p*-Value	AOR	CI (95%)	*p*-value
Regions/Provinces						
Punjab	1					
Sindh	1.02	0.90–1.16	0.73	–	–	–
Baluchistan	1.01	0.84–1.20	0.94	–	–	–
Khyber Pakhtunkhwa	0.82	0.56–1.17	0.27	–	–	–
Gilgit Baltistan	0.55	0.17–1.80	0.32	–	–	–
Islamabad	1.57	0.88–2.78	0.12	–	–	–
Geographical classification						
Rural	1					
Urban	1.09	0.98–1.21	0.11	–	–	–
Respondents age						
15–24 years	1			1		
25–34 years	0.98	0.84–1.15	0.85	0.98	0.84–1.15	0.85
35 years and above	0.76	0.65–0.88	< 0.01	0.78	0.66–0.92	< 0.01
Respondents educational status						
No formal schooling	1			1		
Primary	1.11	0.95–1.30	0.18	1.02	0.86–1.20	0.83
Secondary	1.42	1.24–1.63	< 0.01	1.25	1.06–1.47	< 0.01
Higher	2.42	2.07–2.84	< 0.01	2.05	1.68–2.51	< 0.01
Husbands educational status						
No formal schooling	1			1		
Primary	1.2	0.99–1.47	0.05	1.12	0.92–1.37	0.26
Secondary	1.43	1.22–1.67	< 0.01	1.17	0.99–1.38	0.06
Higher	1.64	1.39–1.93	< 0.01	1.01	0.83–1.22	0.94
Respondents occupation						
Unemployed	1			1		
Professional/Managerial	1.34	1.14–1.57	< 0.01	1.31	1.11–1.54	< 0.01
Agriculture	0.75	0.54–1.05	0.10	1.13	0.79–1.62	0.49
Unskilled workers	0.89	0.70–1.13	0.35	1.07	0.84–1.37	0.56
Husbands occupation						
Unemployed	1			1		
Professional/Managerial	0.99	0.68–1.42	0.95	0.84	0.58–1.23	0.37
Agriculture	0.67	0.45–1.01	0.05	0.67	0.45–1.01	0.05
Unskilled workers	0.74	0.51–1.06	0.10	0.70	0.48–1.02	0.06
Exposure to mass media						
No	1			1		
Yes	1.64	1.34–2.02	< 0.01	1.24	0.99–1.55	0.05
Wealth quintile						
Poorest	1			1		
Poorer	1.27	0.89–1.82	0.17	1.12	0.78–1.61	0.54
Middle	1.49	1.07–2.07	0.01	1.19	0.84–1.69	0.30
Richer	1.36	0.98–1.87	0.06	0.96	0.68–1.36	0.83
Richest	1.86	1.36–2.56	< 0.01	1.09	0.76–1.56	0.62
Respondents autonomy						
Low autonomy	1					
High autonomy	1.04	0.92–1.15	0.53	–	–	–
Respondents attitudes towards wife beating						
Agree on wife beating	1			1		
Disagree on wife beating	1.49	1.32–1.69	< 0.01	1.29	1.13–1.48	< 0.01

The results in Table 3 highlight that respondents from the provinces of Khyber Pakhtunkhwa (AOR = 3.59, 95% CI: 2.20–5.85) and Sindh (AOR = 1.53, 95% CI: 1.30–1.81) were more likely to have high overall knowledge about HIV/AIDS. However, the results showing an association between HIV knowledge and urban/rural locality were not very clear in terms of multivariate logistic regression (OR = 1.86, 95% CI: 1.66–2.08; AOR = 1.04, 95% CI: 0.89–1.21). Here the lower odds ratio in the multivariate model compared to bivariate highlighted the attenuation, which was most likely happened because there were more educated women in urban areas than rural. Findings also revealed that women aged 25–34 years had slightly higher odds of knowledge (AOR = 1.32, 95% CI:1.11–1.56) than those who were 35 years older (AOR = 1.26, 95% CI:1.05–1.52). The odds of comprehensive knowledge were high amongst respondents with secondary (AOR = 1.56, 95% CI: 1.31–1.86) and higher educational status (AOR = 2.59, 95% CI: 2.05–3.27), showing their better knowledge than those with primary education. However, the women working in agriculture sector had lower odds ratio of HIV knowledge (AOR = 0.64, 95% CI: 0.44–0.94) than those who served in professional/managerial positions or unskilled workers. The economic status of respondents showed that the richest respondents were more likely to have high overall knowledge about HIV/AIDS than any other category of economic status. Moreover, respondents who had high autonomy (AOR = 1.15, 95% CI:1.01–1.32) and disagreed with wife beating (AOR = 1.18, 95% CI:1.02–1.37) were more likely to have high overall knowledge about HIV/AIDS.

The results in Table 4 present that odds of positive attitudes towards PLWHAs were high amongst women older than 35 years (AOR = 0.78, 95% CI: 0.66–0.92), having secondary (AOR = 1.25, 95% CI: 1.06–1.47) and higher educational status (AOR = 2.05, 95% CI: 1.68–2.51). The women, who served in professionals/managerial positions (AOR = 1.31, 95% CI: 1.11–1.54) were more likely to express positive attitude than those who worked in agriculture sector. Findings also showed that the women having exposure to mass media (AOR = 1.24; 95% CI: 0.99–1.55) and who disagreed with wife beating (AOR = 1.29, 95% CI: 1.13–1.48) were more likely to have positive attitudes towards PLWHAs.

## Discussion

The present research aimed to explore the associations of various socio-demographic and autonomy-related factors with women’s overall knowledge of HIV/AIDS and attitudes towards PLWHAs in Pakistan. This study highlighted that – despite the high-risk pandemic of HIV/AIDs in Pakistan – only 42% of Pakistani women have ever heard of HIV/AIDS. It showed that more than 50% of women were unaware of HIV/AIDS, raising high concerns. Furthermore, the data revealed that, amongst those women, who heard of HIV/AIDS, the majority (68%) have high overall HIV/AIDS knowledge and more than 55% express positive attitudes towards PLWHAs. These results are consistent with previous research conducted in South India, where most of the respondents had good knowledge about HIV/AIDS and had positive attitudes towards PLWHAs [43]. Similarly, studies conducted in Karachi (Pakistan) [44] and across Pakistan [16] also reported good knowledge about HIV/AIDS among respondents. Nonetheless, these results also highlight the gaps in women’s knowledge and non-discriminatory attitude towards PLWHAs, which needs to be addressed.

Regarding the associated socio-demographic factors, our research further found that overall knowledge of HIV/AIDS is higher amongst women with higher education, residing in urban areas, having exposure to mass media and belonging to the richest wealth quintile. These findings are comparable to studies conducted in India [15, 43], Bangladesh [45] and Ethiopia [40]. Similar results were seen regarding women’s attitudes towards PLWHAs. The findings revealed that the majority of women living in urban areas, having higher education and exposure to mass media had positive attitudes towards PLWHAs. These results are also consistent with research conducted in various African countries [40, 46–48]. These findings taken together suggest a need to target women from rural areas and having no formal schooling through awareness-raising campaigns, particularly using mass media to engage wider audience to enhance their overall knowledge about HIV/AIDS and improve their attitudes towards PLWHAs.

Women’s autonomy is associated with their contribution to decisions related to healthcare utilisation, household and families, while emotional autonomy refers to attitudes towards wife beating. The ability of women to contribute to decisions related to healthcare and emotional well-being has important implications – especially with respect to HIV/AIDS, a disease that impacts women disproportionately. The findings of our research indicate that women with high autonomy and who disagree with wife beating have high overall knowledge and positive attitudes towards PLWHAs. This study revealed that those women who are less educated, with a poor wealth index and less autonomy, are less likely to show positive attitudes towards PLWHAs. These results are also similar to previous research conducted in Ethiopia [40]. This research also endorsed that women’s autonomy is essential to address the impact of disease for improving health outcomes.

Overall, the findings acknowledged the significance of acquiring the factual and comprehensive knowledge regarding causes, prevention and treatment of HIV/AIDS. Knowledge is considered a key to influence individuals’ protective behaviours and eliminating the myths attached to the disease itself and the people who are suffering from it [28, 30, 36]. Therefore, accurate knowledge is instrumental to reduce the various myths and misconceptions related to stigmatisation and negative attitudes associated with HIV/AIDS. These also indicated the prevalent societal and cultural barriers, which prevent the access to comprehensive knowledge. At large, effective educational programmes are required to increase knowledge and alter people’s attitudes and behaviours towards disease, particularly using proven practices of behaviour change, like role models that people can emulate [37, 38, 40]. Various mass media formats, such as TV and radio, could play a pivotal role to gauge wider audience in providing comprehensive and accurate knowledge about HIV/AIDS, particularly in those rural areas where women lack awareness and understanding of the factors associated with AIDS [40, 46]. Similarly, the speeches, talks, seminars and conferences can also be organised for the general audience to overcome the misconceptions attached to AIDS, so that positive attitudes towards PLWHAs can become more widespread. All in all, the research recommends to emphasise the awareness raising campaigns coupled with mass media, which could go a long way to inform and educate people on HIV/AIDS.

### Limitations

Since this research used data from PDHS 2012–13, several limitations arise which are linked to secondary data analyses in general. In particular, due to the cross-sectional study design, no causal relationships can be determined. Further limitations apply to the approach to how knowledge and attitudes were measured.

## Conclusion

The outcomes of this research are encouraging given that women are disproportionately affected by HIV/AIDS. Considering this fact, overall knowledge and positive attitudes are cornerstones of HIV/AIDS prevention, control and treatment. This research supports the significance of women’s autonomy, education and exposure to mass media, to address the disproportionate impact of HIV/AIDS on women. Moreover, high overall knowledge is a key factor in preventing HIV/AIDS as well as in promoting positive attitudes towards PLWHAs in Pakistan.

The researchers conclude that there is a need to increase focused and targeted interventions to enhance women’s knowledge and positive attitudes towards PLWHAs. Efforts are also required to reduce the stigmatisation and negative attitudes towards PLWHAs. This research highlights the role of policymakers, particularly of the National and Provincial AIDS Control Programmes in Pakistan, in ensuring adequate resource planning and implementation of action plans. This could be achieved through launching massive awareness-raising campaigns in local languages for HIV/AIDS prevention, care and support, in which all forms of media, including social media, can be involved. Moreover, there is a need to design explicit awareness-raising messages within the various native languages, keeping in view the cultural norms, especially for rural areas, where outreach workers and local NGOs can be involved. The role of the health sector, particularly of health-facility staff and outreach workers, is also significant. They may also be involved in promoting health education and awareness raising amongst the wider community.

## Additional file

Additional file 1: **Table S3** (a): Multivariate logistics regression of women’s comprehensive HIV/AIDS knowledge with their SES and autonomy from PDHS 2012-2013 (excluding access to information from model). (b): Multivariate logistics regression of women’s comprehensive HIV/AIDS knowledge with their SES and autonomy from PDHS 2012-2013 (excluding wealth index from model). **Table S4** (a): Multivariate logistics regression of women’s attitude towards PLWHAs with their SES and autonomy from PDHS 2012-2013 (excluding access to information from model). (b): Multivariate logistics regression of women’s attitude towards PLWHAs with their SES and autonomy from PDHS 2012-2013 (excluding wealth index from model). (PDF 139 kb)

## Data Availability

Secondary data, available from the Demographic and Health Survey program.

## Abbreviations

AIDS: Acquired Immune Deficiency Syndrome
AOR: Adjusted Odds Ratio
CI: Confidence interval
HIV: Human Immunodeficiency Virus
IMB: Information Motivation Behavioural Skills
NIPS: National Institutes of Population Studies
PDHS: Pakistan Demography and Health Survey
PLWHA: People living with HIV/AIDS
TV: Television
USAID: United States Agency for International Development
