# Supplementary material for: Determinants of overall knowledge of and attitudes towards HIV/AIDS transmission among ever-married women in Pakistan: evidence from the Demographic and Health Survey 2012–13
Source: BMC Public Health. 2019 Jun 21;19:793. doi: 10.1186/s12889-019-7124-3 (PMC6588857; doi:10.1186/s12889-019-7124-3)
Supplement: Supplementary file 1 — Table S3 (a): Multivariate logistics regression of women’s comprehensive HIV/AIDS knowledge with their SES and autonomy from PDHS 2012-2013 (excluding access to information from model). (b): Multivariate logistics regression of women’s comprehensive HIV/AIDS knowledge with their SES and autonomy from PDHS 2012-2013 (excluding wealth index from model). Table S4 (a): Multivariate logistics regression of women’s attitude towards PLWHAs with their SES and autonomy from PDHS 2012-2013 (excluding access to information from model). (b): Multivariate logistics regression of women’s attitude towards PLWHAs with their SES and autonomy from PDHS 2012-2013 (excluding wealth index from model). (PDF 139 kb) [file 12889_2019_7124_MOESM1_ESM.pdf]

Additional file 1

Table S3(a): Multivariate logistics regression of women's comprehensive HIV/AIDS knowledge with their SES and autonomy from PDHS 2012-2013 (excluding access to information from model)

**Characteristics**	**Women's comprehensive HIV/AIDS knowledge**		
	**Multivariate**		
	**AOR**	**CI (95%)**	**p-value**
**Regions/Provinces**			
Punjab	1		
Sindh	1.54*	1.31-1.82	**<0.01**
Baluchistan	1.16	0.94-1.43	0.14
Khyber Pakhtunkhwa	3.51*	2.16-5.71	**<0.01**
Gilgit Baltistan	1.38	0.34-5.51	0.64
Islamabad	1.59	0.74-3.41	0.23
**Geographical classification**			
Rural	1		
Urban	1.04	0.89-1.21	0.58
**Respondents age**			
15-24 years	1		
25-34 years	1.31*	1.10-1.55	**<0.01**
35 years and above	1.24*	1.03-1.50	**0.02**
**Respondents educational status**			
No formal schooling	1		
Primary	1.15	0.96-1.38	0.11
Secondary	1.58*	1.33-1.89	**<0.01**
Higher	2.63*	2.08-3.33	**<0.01**
**Husbands educational status**			
No formal schooling	1		
Primary	1.01	0.81-1.24	0.96
Secondary	1.06	0.89-1.27	0.48
Higher	1.17	0.94-1.45	0.14
**Respondents occupation**			
Unemployed	1		
Professional/Managerial	1.11	0.85-1.45	0.43
Agriculture	1.22	0.90-1.66	0.19
Unskilled workers	0.72	0.46-1.11	0.14
**Wealth quintile**			
Poorest	1		
Poorer	0.98	0.67-1.43	0.92
Middle	1.00	0.69-1.43	0.99
Richer	1.03	0.71-1.49	0.86
Richest	1.70*	1.15-2.51	**0.01**
**Respondents autonomy**			
Low autonomy	1		
High autonomy	1.16*	1.02-1.33	**0.02**
**Respondents attitudes towards wife beating**			
Agree on wife beating	1		
Disagree on wife beating	0.84*	0.73-0.97	**0.02**

Table S3(b): Multivariate logistics regression of women's comprehensive HIV/AIDS knowledge with their SES and autonomy from PDHS 2012-2013 (excluding wealth index from model)

Characteristics	Women's comprehensive HIV/AIDS knowledge		
	Multivariate		
	AOR	CI (95%)	p-value
**Regions/Provinces**			
Punjab	1		
Sindh	1.53*	1.30-1.81	**<0.01**
Baluchistan	1.16	0.94-1.43	0.15
Khyber Pakhtunkhwa	3.39*	2.08-5.52	**<0.01**
Gilgit Baltistan	1.11	0.28-4.44	0.87
Islamabad	1.76	0.82-3.76	0.14
**Geographical classification**			
Rural	1		
Urban	1.24*	1.09-1.42	**<0.01**
**Respondents age**			
15-24 years	1		
25-34 years	1.34*	1.13-1.59	**<0.01**
35 years and above	1.35*	1.12-1.63	**0.01**
**Respondents educational status**			
No formal schooling	1		
Primary	1.15	0.96-1.37	0.11
Secondary	1.66*	1.40-1.97	**<0.01**
Higher	3.00*	2.38-3.77	**<0.01**
**Husbands educational status**			
No formal schooling	1		
Primary	1.03	0.83-1.28	0.75
Secondary	1.14	0.95-1.36	0.15
Higher	1.33*	1.08-1.63	**0.01**
**Respondents occupation**			
Unemployed	1		
Professional/Managerial	1.16	0.89-1.52	0.26
Agriculture	1.21	0.89-1.64	0.21
Unskilled workers	0.75	0.48-1.17	0.20
**Exposure to mass media**			
No	1		
Yes	1.30*	1.03-1.64	**0.02**
**Respondents autonomy**			
Low autonomy	1		
High autonomy	1.13	0.99-1.29	0.06
**Respondents attitudes towards wife beating**			
Agree on wife beating	1		
Disagree on wife beating	0.81*	0.70-0.94	**<0.01**

Table S4(a): Multivariate logistics regression of women's attitude towards PLWHAs with their SES and autonomy from PDHS 2012-2013 (excluding access to information from model)

Characteristics	Women's attitude towards PLWHAs		
	Multivariate		
	**AOR**	**CI (95%)**	**p-value**
**Respondents age**			
15-24 years	1		
25-34 years	0.98	0.84-1.15	0.80
35 years and above	0.77*	0.65-0.91	**<0.01**
**Respondents educational status**			
No formal schooling	1		
Primary	1.03	0.87-1.22	0.71
Secondary	1.27*	1.08-1.49	**<0.01**
Higher	2.08*	1.70-2.55	**<0.01**
**Husbands educational status**			
No formal schooling	1		
Primary	1.13	0.83-1.28	0.23
Secondary	1.18*	0.95-1.36	**0.05**
Higher	1.01	1.08-1.63	0.90
**Respondents occupation**			
Unemployed	1		
Professional/Managerial	0.93	0.73-1.19	0.57
Agriculture	1.23	0.93-1.63	0.15
Unskilled workers	1.06	0.69-1.61	0.79
**Husbands occupation**			
Unemployed	1		
Professional/Managerial	0.84	0.58-1.23	0.37
Agriculture	0.67	0.45-1.01	**0.05**
Unskilled workers	0.70	0.48-1.02	0.06
**Wealth quintile**			
Poorest	1		
Poorer	1.14	0.79-1.64	0.47
Middle	1.24	0.88-1.75	0.21
Richer	1.01	0.71-1.42	0.96
Richest	1.15	0.81-1.64	0.43
**Respondents attitudes towards wife beating**			
Agree on wife beating	1		
Disagree on wife beating	0.79*	0.67-0.88	**<0.01**

Table S4(b): Multivariate logistics regression of women's attitude towards PLWHAs with their SES and autonomy from PDHS 2012-2013 (excluding wealth index from model)

Characteristics	Women's attitude towards PLWHAs		
	Multivariate		
	**AOR**	**CI (95%)**	**p-value**
**Respondents age**			
15-24 years	1		
25-34 years	0.98	0.84-1.15	0.84
35 years and above	0.78*	0.66-0.91	**0.02**
**Respondents educational status**			
No formal schooling	1		
Primary	1.01	0.85-1.19	0.92
Secondary	1.22*	1.05-1.43	**0.01**
Higher	2.05*	1.68-2.48	**<0.01**
**Husbands educational status**			
No formal schooling	1		
Primary	1.12	0.92-1.37	0.25
Secondary	1.17	0.99-1.38	**0.05**
Higher	1.01	0.84-1.23	0.90
**Respondents occupation**			
Unemployed	1		
Professional/Managerial	0.92	0.72-1.17	0.50
Agriculture	1.20	0.91-1.59	0.19
Unskilled workers	1.04	0.69-1.58	0.83
**Husbands occupation**			
Unemployed	1		
Professional/Managerial	0.84	0.58-1.22	0.36
Agriculture	0.67*	0.45-1.01	**0.05**
Unskilled workers	0.70	0.48-1.02	0.06
**Exposure to mass media**			
No	1		
Yes	1.23*	0.99-1.53	**0.05**
**Respondents attitudes towards wife beating**			
Agree on wife beating	1		
Disagree on wife beating	0.78*	0.68-0.89	**<0.01**
